# The effectiveness of chemotherapy for patients with pT3N0M0 renal pelvic urothelial carcinomas: An inverse probability of treatment weighting comparison using Surveillance, Epidemiology, and End Results data

**DOI:** 10.1002/cam4.3238

**Published:** 2020-06-25

**Authors:** Zefu Liu, Jialing Huang, Xiangdong Li, Chaowen Huang, Yunlin Ye, Jinxin Zhang, Zhouwei Liu

**Affiliations:** ^1^ Department of Urology Cancer Center Sun Yat‐sen University Guangzhou China; ^2^ Department of Medical Statistics School of Public Health Sun Yat‐Sen University Guangzhou China

**Keywords:** chemotherapy, upper urinary tract, urothelial carcinoma

## Abstract

**Introduction:**

Unlike the established evidence to use chemotherapy for urothelial carcinoma of the bladder, presently there are insufficient data to inform a recommendation on upper urinary tract urothelial carcinoma treatment. The prognosis for patients with stage T4 and positive lymph nodes is poor; however, primary tumors in the renal pelvis are associated with favorable prognoses compared to those located in the ureter. Our study aimed at investigating the effectiveness of chemotherapy in patients with pT3N0M0 renal pelvic urothelial carcinomas (RPUC) who have relative favorable prognosis.

**Methods:**

Patients with pT3N0M0 tumors who underwent radical nephroureterectomy combined with bladder cuff excision between 2005 and 2014 and registered in the Surveillance, Epidemiology, and End Results database were eligible for inclusion (n = 939). Baseline characteristics between the chemotherapy and observation groups were controlled for with inverse probability of treatment weighting (IPTW)‐adjusted analysis.

**Results:**

After the IPTW‐adjusted analysis, the 5‐year IPTW‐adjusted rates of overall survival (OS) for the chemotherapy and observation groups were 53.1% and 44.9%, respectively. The IPTW‐adjusted Kaplan‐Meier curves suggested that chemotherapy was associated with increased OS compared with observation (*P* = .028). In the IPTW‐adjusted Cox proportional hazards regression model, chemotherapy was associated with favorable survival benefits compared with observation (hazard ratio [HR] 0.71, 95% CI 0.52‐0.92, *P* = .031), and this was maintained after bootstrapping (HR 0.72, 95% CI 0.49‐0.93). Chemotherapy had a protective effect on OS benefits, which were found in a majority of the results of the subgroup analysis and were consistent with the main results (all *P*‐interactions > 0.05).

**Conclusion:**

Chemotherapy may provide significant OS benefits for patients with pT3N0M0 RPUC. The results of our study could strengthen the evidence for using adjuvant chemotherapy in this rare group of patients.

## Graphical abstract

Chemotherapy appears to provide significant overall survival benefits for patients with pT3N0M0 renal pelvic urothelial carcinomas. Our results showed that the effectiveness of chemotherapy is not related to the tumor size and pattern of invasion. The findings of our study could strengthen the evidence for using adjuvant chemotherapy for this rare group of patients.

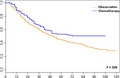

## 1 INTRODUCTION

Upper urinary tract urothelial carcinomas (UTUC) are rare, accounting for 5%‐10% of all urothelial carcinomas.^1^, ^2^ Much of the current clinical decision‐making in UTUCs uses a guideline based on urothelial carcinomas of the bladder.^1^ However, unlike the established evidence based on chemotherapy for urothelial carcinoma of the bladder, there are insufficient data to inform recommendations for UTUC treatment.^2^ Previous retrospective studies of adjuvant chemotherapy yielded conflicting results and had low statistical power.^3^, ^4^, ^5^, ^6^, ^7^, ^8^, ^9^ The results of the POUT clinical trial recently supported the benefit of administering adjuvant chemotherapy for patients undergoing RNU and provided the strongest evidence for using adjuvant chemotherapy in the standard of care of these patients, with improved disease‐free survival (DFS, hazard ratio [HR] = 0.45, *P* = .0002) obtained with chemotherapy.^10^ However, the use of DFS as a surrogate endpoint for overall survival (OS) is still controversial.^11^ Therefore, outcomes of OS supported by more evidence are still awaited.

In the POUT clinical trial, 91% of the patients had negative lymph nodes, and the subgroup analysis showed that the clear benefit was attributable to these patients.^10^ Nevertheless, in the subgroup analysis, the improved DFS did not reach significance in patients with positive lymph nodes, due to their small sample size.^10^ However, in the real world, a study based on National Cancer Data Base (NCDB) showed that patients with lymph node metastases and tumors located in the ureter had a higher possibility of receiving adjuvant chemotherapy than patients with negative lymph nodes and tumors located in the renal pelvis.^3^ Furthermore, a study based on an international UTUC database collaboration between Europe and the United States showed that patients who receive adjuvant chemotherapy harbored more advanced disease.^4^, ^7^ Undoubtedly, the prognosis for patients with T4 stage, lymph‐node‐positive disease is poor, and serious consideration is given to providing adjuvant chemotherapy following radical nephroureterectomy for these patients.^3^, ^7^ Currently, only 16.2%‐35.1% of pT3N0M0 patients receive adjuvant chemotherapy.^5^, ^7^ The EAU guideline considered that primary tumors in the renal pelvis were associated with favorable prognosis compared with those located in the ureter.^2^, ^5^, ^12^ In summary, it was reported that a subgroup of patients with pT3N0M0 renal pelvic urothelial carcinomas (RPUC) had a better prognosis, but lower percentage of receiving chemotherapy, than those with locally advanced UTUC. Importantly, less is known about the relationship between chemotherapy and survival in patients with pT3N0M0 RPUC after a radical nephroureterectomy, and there are conflicting results with largely retrospective studies. The effectiveness of chemotherapy for the OS for patients with pT3N0M0 RPUCs merits study. Therefore, we investigated the effects of chemotherapy using data from the Surveillance, Epidemiology, and End Results (SEER) database, which will address the use of adjuvant chemotherapy in patients with pT3N0M0 RPUC.

## 2 METHODS

### 2.1 Study population

**Figure 1:**
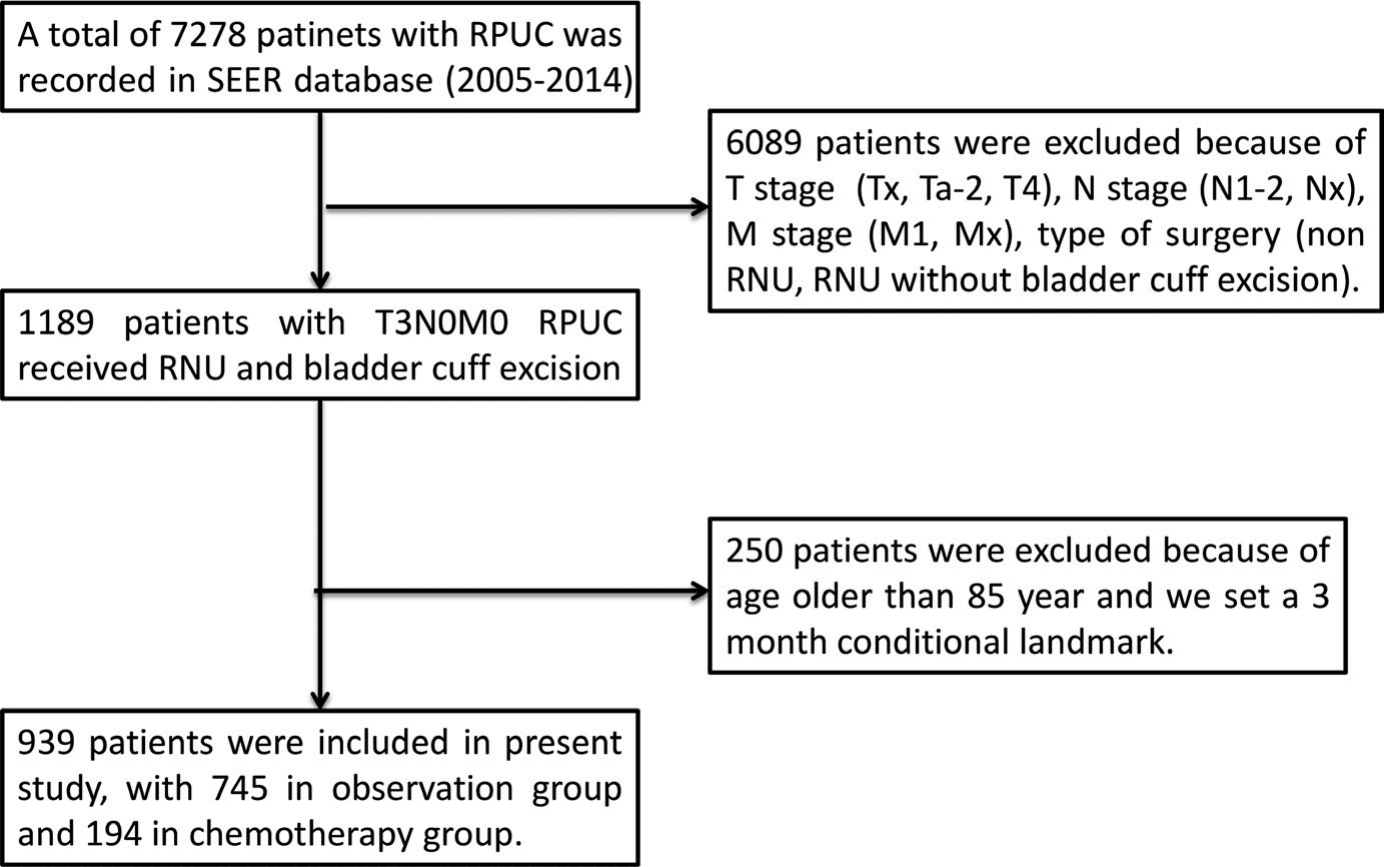
Study flow. RPUC: renal pelvic urothelial carcinomas, RNU: radical nephroureterectomy

The SEER database collects data from 17 cancer registries, covering approximately 28% of the US population. It classifies pathological features according to the International Classification of Disease for Oncology (ICD‐O‐3) histology codes. A total of 7278 cases of tumors located in the renal pelvis (code C65.9) were added to the SEER database between 2005 and 2014. Patients with transitional cell carcinomas (codes 8120/3, 8122/3, 8130/3, and 8131/3) were eligible for inclusion. Patients who underwent radical nephroureterectomy combined with bladder cuff excision (surgery primary site code 40) and whose disease was subsequently characterized as pathological TNM stage T3N0M0 were included in the study (Figure 1). Patients older than 85 years were excluded because they were less likely to receive chemotherapy. To determine renal peripelvic fat invasion (RFI) and renal parenchymal invasion (RPI), CS Extension codes (2004‐2014) were used. OS was defined as the time from the date of surgery to the date of death due to any cause and was defined according to the vital status recode. Sequences of chemotherapy were not reported in the SEER database. A 3‐month conditional landmark analysis was used to remove bias relating to early mortality.

### 2.2 Statistical analysis

Baseline characteristics between the chemotherapy and observation groups were controlled for with inverse probability of treatment weighting (IPTW)‐adjusted analyses.^13^ Propensity score estimation relied on the inclusion of all observed covariates into a multivariate logistic regression model in which treatment assignment (chemotherapy vs observation) was regressed based on age, gender, race, tumor size, grade, type of invasion (RPI or RFI), number of lymph nodes removed, and insurance status. A multivariable logistic regression model used to estimate propensity scores is reported. The estimated propensity scores were used to calculate stabilized IPTW average treatment effect weights.^13^ The covariate balance between the chemotherapy and observation groups was evaluated using Austin's method of calculating the standardized difference.^14^ Imbalance between the two groups was defined as a standard difference of greater than 10%. The primary endpoint (OS) was compared using an adjusted Kaplan‐Meier estimator and log‐rank test with IPTW.^15^ We also used a weighted Cox proportional hazards regression model to calculate the IPTW‐adjusted hazard HR of the chemotherapy vs observation groups, and a bootstrap approach was used to assess the impact of variance variability using 1000 random datasets that were drawn randomly to replace the original dataset. We performed exploratory analyses to evaluate the IPTW‐adjusted hazard ratios (chemotherapy vs observation) categorized by age (<75 and ≥75 years), gender (male or female), race (white, black, and other), insurance status (yes, no/unknown), type of invasion (RPI or RFI), number of lymph nodes removed (none, 1‐7, and >8), tumor size (≤3 cm, >3 cm, and unknown), and grade (G1‐2, G2‐3, and unknown).^14^ We combined the type of invasion and tumor size to create three prognosis groups: RPI and ≤3 cm, RPI and >3 cm, RFI and any size. Moreover, we calculated the adjusted HR of chemotherapy vs observation by performing multivariate Cox regression analysis in each subgroup. In this multivariate subgroup analysis, we treated the other confounding factors as covariates and adjusted their effects. For lymph node dissection and insurance status, we did not calculate the HR because of the limitation imposed by the relatively small sample size in each subgroup. Statistical analysis was carried out using R software version 3.4.2 (Vienna, Austria). All tests were two‐sided and *P* < .05 was considered statistically significant. The R packages IPWsurvival, survival, survey, tableone, forestplot, and boot were applied for the respective data analyses.

## 3 RESULTS

### 3.1 Comparison of baseline characteristics

**Figure 2:**
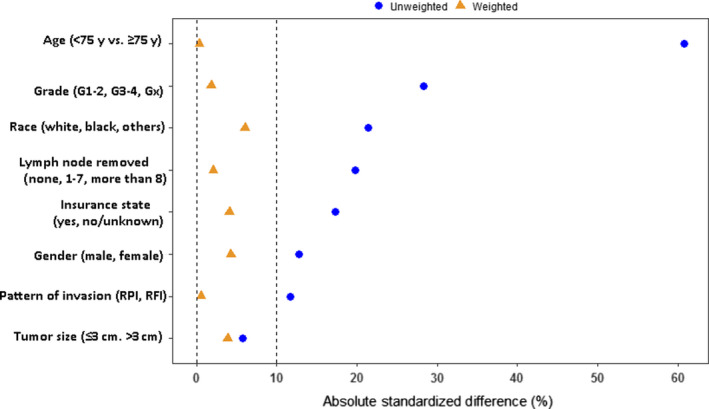
Effect of inverse probability of treatment weighting. RPI: renal parenchymal invasion, RFI: renal peripelvic fat invasion

**Figure 3:**
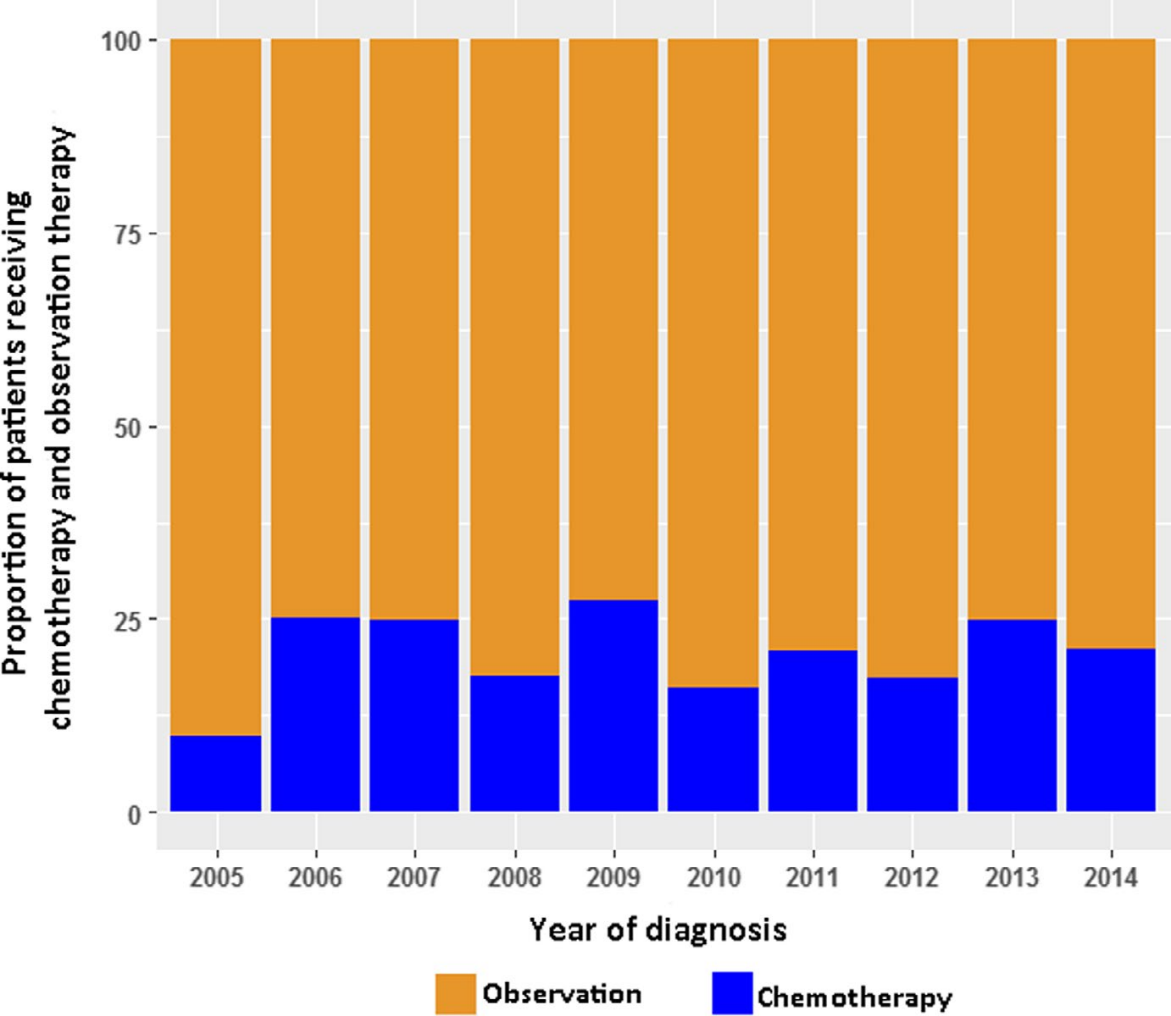
Use of chemotherapy vs observation for patients who underwent radical nephroureterectomy from the SEER database between 2005 and 2014

The study flow is presented in Figure 1. Table 1 presents the baseline characteristics of the study population, which was divided into chemotherapy and observation groups. There was significant heterogeneity between the groups in characteristics such as gender, age, race, pattern of invasion (RFI and RPI), grade, number of lymph nodes removed, and insurance status (standard difference >10%, Table 1). The highest and the lowest weights in the chemotherapy group 4.28 and 0.34, respectively. The corresponding values in the non‐chemotherapy group were 1.72 and 0.81. Patients who received chemotherapy were younger, more likely to have insurance, had higher grade tumors, were more likely to have RFI, and were more likely to have lymph node dissection. In addition, males and non‐whites had a higher probability of receiving chemotherapy. After the IPTW‐adjusted analysis, the two groups were comparable and all standardized differences were less than 10% (Table 2). The effective sample sizes after IPTW were 193.6 in the chemotherapy group and 744.7 in the control group, respectively (Table 2). The results obtained with the multivariable logistic regression model used to estimate propensity scores are reported in Table 3. Unweighted and weighted baseline characteristics of patients stratified by treatment group are shown in Figure 2. Figure 3 shows the proportion patients receiving chemotherapy vs observation over the period 2005‐2014.

**Table 1:** Comparison of baseline characteristics before inverse probability of treatment weighting‐adjusted analysis

	Chemotherapy	Observation	Standard difference
Patients (n)	194	745	‐
Gender (%)			
Male	125 (64.4)	434 (58.3)	0.127
Female	69 (35.6)	311 (41.7)	
Age			
<75 years	160 (82.5)	414 (55.6)	0.608
≥75 years	34 (17.5)	331 (44.4)	
Race			
White	160 (82.5)	666 (89.4)	0.213
Black	16 (8.2)	29 (3.9)	
Others	18 (9.3)	50 (6.7)	
Patterns of invasion			
RPI	121 (62.4)	506 (67.9)	0.117
RFI	73 (37.6)	239 (32.1)	
Tumor size			
≤3 cm	65 (33.5)	262 (35.2)	0.057
>3 cm	118 (60.8)	434 (58.3)	
Unknown	11 (5.7)	49 (6.6)	
Grade			
G1‐2	12 (6.2)	106 (14.2)	0.283
G3‐4	165 (85.1)	596 (80.0)	
Unknown	17 (8.8)	43 (5.8)	
Lymph node removed (n)			
None	145 (74.7)	599 (80.4)	0.198
1‐7	31 (16.0)	113 (15.2)	
≥8	18 (9.3)	33 (4.4)	
Insurance			
Yes	167 (86.1)	593 (79.6)	0.173
No/unknown	27 (13.9)	152 (20.4)	

Note

RFI: renal peripelvic/periureteral fat invasion; RPI: renal parenchymal invasion; n: number.

**Table 2:** Comparison of baseline characteristics after inverse probability of treatment weighting‐adjusted analysis

	Chemotherapy	Observation	Standard difference
Patients (n)	193.6	744.7	‐
Gender (%)			
Male	119.3 (61.6)	443.5 (59.6)	0.042
Female	74.3 (38.4)	301.2 (40.4)	
Age			
<75 years	118.6 (61.3)	455.0 (61.1)	0.003
≥75 years	75.0 (38.7)	289.7 (38.9)	
Race			
White	174.0 (89.9)	656.6 (88.2)	0.060
Black	8.7 (4.5)	35.6 (4.8)	
Others	10.9 (5.6)	52.5 (7.0)	
Patterns of invasion			
RPI	128.8 (66.5)	497.5 (66.8)	0.006
RFI	64.8 (33.5)	247.2 (33.2)	
Tumor size			
≤3 cm	68.4 (35.3)	259.3 (34.8)	0.039
>3 cm	114.7 (59.2)	438.1 (58.8)	
Unknown	10.5 (5.4)	47.3 (6.3)	
Grade			
G1‐2	23.2 (12.0)	93.6 (12.6)	0.018
G3‐4	158.2 (81.7)	603.6 (81.1)	
Unknown	12.2 (6.3)	47.5 (6.4)	
Lymph node removed (n)			
None	152.9 (79.0)	589.5 (79.2)	0.021
1‐7	31.0 (16.0)	115.1 (15.5)	
≥8	9.7 (5.0)	40.1 (5.4)	
Insurance			
Yes	159.8 (82.6)	603.1 (81.0)	0.041
No/unknown	33.8 (17.4)	141.6 (19.0)	

Note

RFI: renal peripelvic/periureteral fat invasion; RPI: renal parenchymal invasion; n: number.

**Table 3:** Multivariable logistic regression model used to estimate propensity scores

Term	Estimate	SE	Statistic	*P*‐value	OR	95% CI
Intercept	−1.986	0.353	−5.623	<0.001	0.137	0.066‐0.265
Gender						
Male					Reference	
Female	−0.223	0.177	−1.261	0.207	0.800	0.563‐1.129
Age						
<75 years					Reference	
≥75 years	−1.305	0.206	−6.319	<0.001	0.271	0.179‐0.402
Race						
White					Reference	
Black	0.685	0.341	2.007	0.045	1.985	0.998‐3.838
Others	0.385	0.308	1.249	0.212	1.469	0.786‐2.648
Lymph node removed						
None					Reference	
1‐7	0.037	0.233	0.159	0.874	1.038	0.650‐1.621
More than 8	0.580	0.324	1.788	0.074	1.786	0.931‐3.342
Grade						
G1‐2					Reference	
G3‐4	1.002	0.326	3.075	0.002	2.722	1.492‐5.404
Gx	1.296	0.436	2.975	0.003	3.656	1.569‐8.762
Tumor size						
≤3 cm					Reference	
>3 cm	0.084	0.183	0.457	0.647	1.087	0.762‐1.561
Unknown	−0.172	0.376	−0.458	0.647	0.842	0.386‐1.709
Pattern of invasion						
RPI					Reference	
RFI	0.338	0.177	1.906	0.057	1.402	0.988‐1.981
Insurance						
Yes					Reference	
No/Unknown	−0.470	0.237	−1.980	0.048	0.625	0.386‐0.982

Note

RPI: renal parenchymal invasion; RFI: renal peripelvic/periureteral fat invasion.

### 3.2 Survival analysis

**Figure 4:**
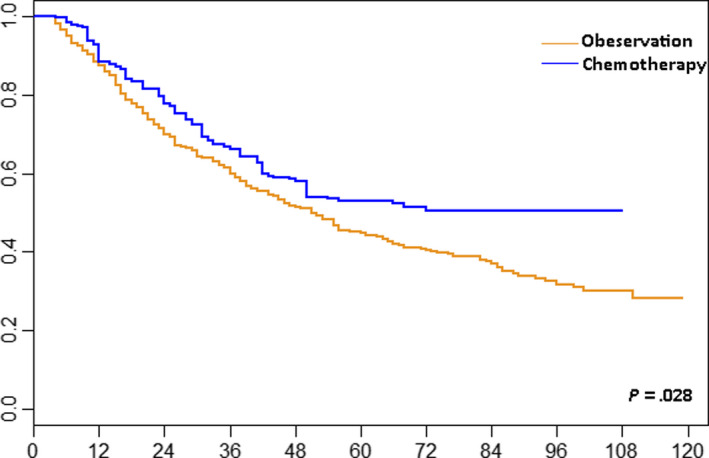
Inverse probability of treatment weighting‐adjusted Kaplan‐Meier analysis of overall survival in patients receiving chemotherapy vs observation

**Figure 5:**
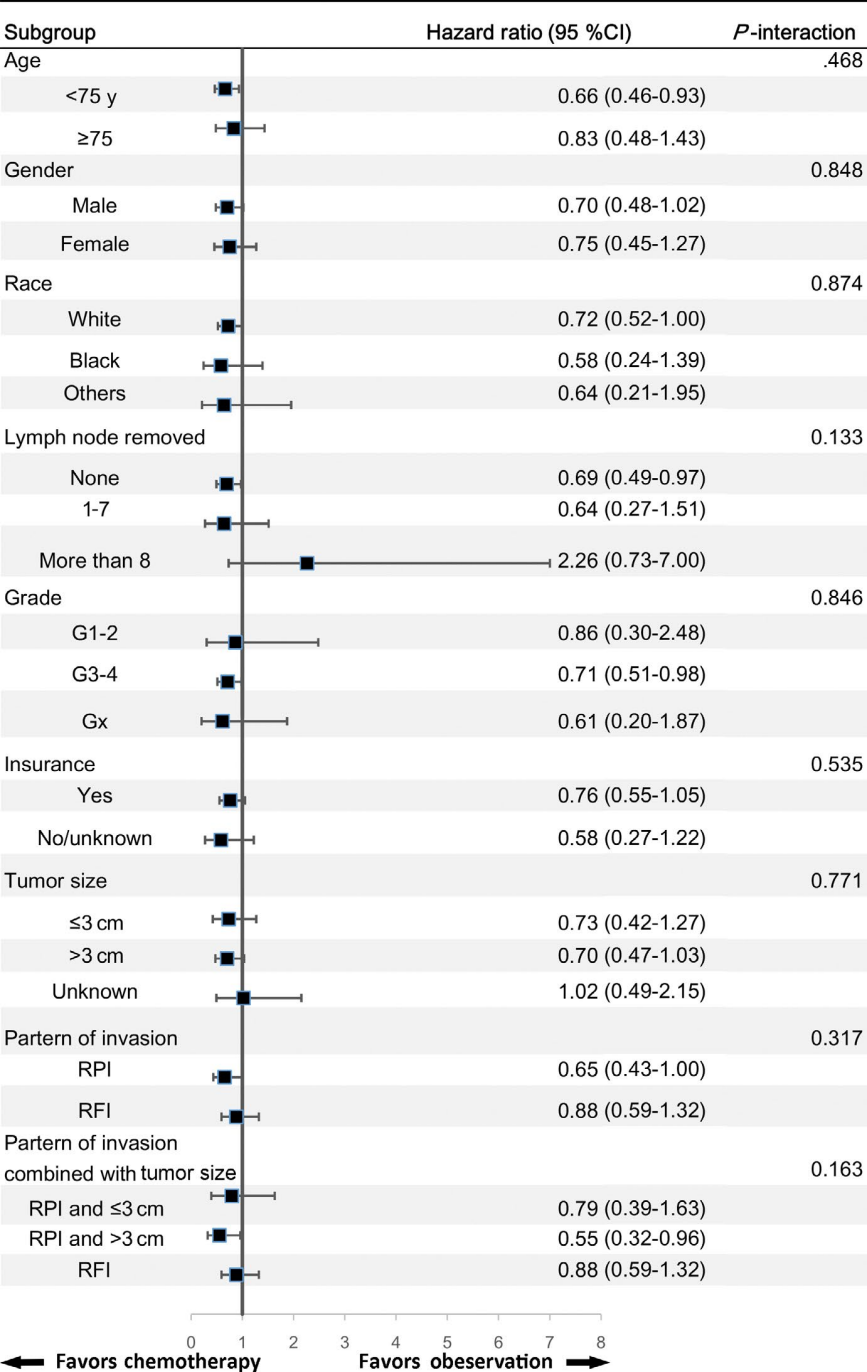
Inverse probability of treatment weighting‐adjusted hazard ratios of chemotherapy vs observation for patients receiving radical nephroureterectomy depicted by a Forrest plot. RPI: renal parenchymal invasion, RFI: renal peripelvic fat invasion

The median follow‐up times for the chemotherapy and observation groups were 60 months (95% confidence interval [CI] 48‐69) and 54 months (95% CI 50‐58), respectively. The 5‐year IPTW‐adjusted rates of OS for the chemotherapy and observation groups were 53.1% and 44.9%, respectively. IPTW‐adjusted Kaplan‐Meier curves (Figure 4) suggested that chemotherapy was associated with increased OS compared with observation (*P *= .028). In the IPTW‐adjusted Cox proportional hazards regression model, chemotherapy was associated with favorable survival benefits compared with observation (HR = 0.71, 95% CI 0.52‐0.92, *P *= .031), and this was maintained after bootstrapping (HR = 0.72, 95% CI 0.49‐0.93). Subgroup analysis by type of invasion (RPI or RFI), age, gender, race, number of lymph nodes removed, tumor size, and grade was noteworthy (Figure 5). The chemotherapy group showed a protective effect in terms of OS benefits, which appeared in a majority of the results of subgroup analysis and was consistent with our main results (all *P* > .05). We noticed that the number of patients with tumor size unknown and lymph dissection more than 8 was only 51 and 34, which contributed to the potentially unstable effect size estimates. Furthermore, it was possible to classify patients into three prognostic groups: RPI and ≤3 cm, RPI and >3 cm, and PFI and any size. The increased rate of OS with chemotherapy was consistent in these subgroups (RPI and ≤3 cm: HR = 0.79, 95% CI 0.39‐1.63; RPI and >3 cm: HR = 0.55, 95% CI 0.32‐0.96; PFI and any size: HR = 0.88, 95% CI 0.59‐1.32, Figure 5). In addition, we calculated the adjusted HR of chemotherapy vs observation by multivariate Cox regression analysis in each subgroup (Table S1). In this multivariate subgroup analysis, we treated the other confounding factors as covariates and adjusted their effects. These results were also consistent with the results calculated by IPTW.

## 4 DISCUSSION

Our data derived from a real‐world database (SEER database) validate the benefits of using chemotherapy in patients with pT3N0M0 RPUC, especially benefits related to OS. Furthermore, we noticed that some differences might be weakened by the relatively small sample sizes in subgroup analysis, but no heterogeneity of OS benefits was detected across a majority of the subgroups. Moreover, the adjusted HR of chemotherapy vs observation by multivariate COX regression was consistent with the adjusted HR obtained by IPTW. The *P*‐values for the interaction effects were calculated by testing the interaction terms in the respective interaction models in the whole sample (n = 939). We believed the results from the interaction models were more reliable compared with those of the subgroup analyses due to the sufficient power in the interaction models. This showed that there were no significant interaction effects between the chemotherapy and the covariates, which further supports our main finding.

The better effect of adjuvant chemotherapy for high‐risk patients with negative lymph node was seen in a subgroup analysis of the NCDB study (pT3‐4N0: HR = 0.69, 95% CI: 0.57‐0.84)^3^ and the POUT clinical trial (pT2‐4N0: HR = 0.40, 95% CI: 0.25‐0.63).^10^ The POUT clinical trial primarily recruited high‐risk patients with N0 (91%) UTUC and did provide the strongest evidence for using adjuvant chemotherapy in the standard of care of these patients.^10^ In our study, we focused on patients with pT3N0M0 RPUC who showed a relatively favorable prognosis among those with pT3‐4 or N+ tumors. Importantly, according to analyses of some large multi‐institutional databases, pT3N0‐2 tumors account for approximately 22.1%‐37.0% of all UTUCs ^8^, ^16^, ^17^, ^18^, ^19^ and 31.0%‐43.0% of all renal pelvic tumors.^16^, ^19^, ^20^ Patients with pT3N0M0 RPUCs appear to account for almost a third of patients with UTUC. However, as with most rare cancers, their treatment is based on a relatively small body of literature. Currently, only 18.7% of patients with pT3N0M0 RPUC receive adjuvant chemotherapy.^5^ The findings of our study could strengthen the evidence for using adjuvant chemotherapy for this rare group of patients.

Furthermore, we noticed that some retrospective data for adjuvant chemotherapy are conflicting. Seisen et al^3^ and Haung et al^5^ observed that there were survival benefits of using adjuvant chemotherapy for pT3‐4N0M0 UTUC in the period 2004‐2012, and for pT3N0M0 tumors from 2004 to 2014, respectively. Conversely, a stage‐to‐stage comparison from 1992 to 2006 using an international UTUC database did not favor adjuvant chemotherapy in pT3N0M0 stage disease (HR = 1.14, *P* = .529),^7^ and when the sample size was increased there even appeared to be an increased risk of overall mortality (HR = 1.47, *P* = .172) in patients with pT3‐4N0M0 (from 2000 to 2015) stage disease.^4^ In fact, their results showed that in patients without lymph node metastases, adjuvant chemotherapy was associated with an increased risk of overall mortality.^4^, ^7^ It is likely that the positive effect of chemotherapy seen in our study and that of Seisen et al and Huang et al ^3^, ^5^ is due to the more recent timeframe, improved radical nephroureterectomy techniques, more efficient cisplatin‐based chemotherapy regimens, and more extensive lymph node dissection. The different sources of data in studies based on international databases (obtained from community oncology)^4^ and the NCDB database^3^ (obtained from high‐volume hospitals) may contribute to conflicting outcomes for adjuvant chemotherapy. Although the overlap of the NCDB and SEER databases is significant, the data included in NCDB are based on the hospital system whereas the data in the SEER database are based on the population. Not all results derived from the NCDB study could be generalized to the entire US.^21^ In addition, the use of chemotherapy may be limited because of the post‐RNU drop‐off in half renal function in clinical practice. In this context, our IPTW‐adjusted analysis based on the SEER database showed a significant OS benefit of chemotherapy, and these outcomes may facilitate the clinical practice changes for pT3N0M0 RPUC.

Previous studies have reported that 4.7%‐45.8% of pT3N0‐2 UTUC tumors are low‐grade,^5^, ^20^, ^22^, ^23^ and the proportion of low‐grade tumors receiving chemotherapy is reported as 6.4%‐50.0%^4^, ^5^, ^7^; less is known about the efficacy of chemotherapy in these patients. Our study suggests that chemotherapy for pT3N0M0 patients with low‐grade (G1‐2) tumors is also beneficial in terms of survival benefit (HR = 0.86, 95% CI 0.30‐2.48), but difference might be weakened by the relatively small sample sizes. Two previously published analyses using propensity scoring did not incorporate lymph node dissection into the model.^3^, ^4^ The outcomes of the POUT clinical trial validate the survival benefits in patients with pN0, and whether the use of more extensive lymph node dissection would offer additional benefits is uncertain. Therefore, we considered lymph node dissection as a potential confounder. We found that chemotherapy provided a significant survival benefit in patients who underwent no lymph node dissection and a trend of benefits in patients who had undergone lymph node removal (harvested from 1 to 7). This outcome could be attributed to the “Will Rogers” stage migration phenomenon, whereby more extensive lymphadenectomy could lead to more accurate staging. In the real world, patients with positive lymph nodes might be the most suitable candidates for receiving adjuvant chemotherapy and showed clear survival benefits in some retrospective studies.^3^ Certainly, the necessity of lymphadenectomy and the optimum degree of lymph node removal is still under debate.^24^ According to Kondo et al^25^ and Roscigno et al,^26^ a lymph node count of at least eight during lymphadenectomy favorably influenced survival. Therefore, in our study, removal of eight lymph nodes was considered as the cutoff value in predicting OS. Although chemotherapy was not associated with benefits in improving OS for patients who had undergone removal of more than eight lymph nodes, we think that this result might be attributable to the low statistical power caused by the small sample size.

For patients who underwent radical removal of half renal units after undergoing radical nephroureterectomy, the success of the POUT clinical trial give us an opportunity to consider the neoadjuvant setting. The ongoing trial, URANUS, will address the effectiveness of neoadjuvant chemotherapy in comparison with adjuvant chemotherapy. PD‐1/PD‐L1 checkpoint inhibitors may be a better choice of adjuvant treatment, with their low toxicity, high compliance, and longer response duration.^27^, ^28^ In addition, concomitant or sequential administration of PD‐1/PD‐L1 checkpoint inhibitors and chemotherapy possibly further enhances their efficacy,^29^ by inhibiting tumors’ evasion of the immune response and by increasing sensitivity to chemotherapy. Robinson et al conducted a study based on an integrated analysis of whole‐exome and RNA‐sequencing of UTUC, and the outcome showed that most of the UTUCs were categorized to luminal‐papillary and T‐cell depleted. Moreover, sporadic UTUCs contain a lower total mutational burden than urothelial carcinomas of the bladder.^30^ Most importantly, they indicated that FGFR3 inhibition potentially remolds the immune contexture of UTUC and provides a rationale for combined treatment with PD‐1/PD‐L1 and an FGFR3 inhibitor.^30^ Recently, a pan‐FGFR inhibitor was also established for advanced urothelial carcinomas with FGFR alterations, including UTUC.^31^ However, data for combined chemotherapy and immunotherapy and/or FGFR inhibitors are unmet needs in the clinical context.

Some investigators have attempted to stratify patients with pT3 stage RPUC into heterogeneous prognosis groups, for example microscopic inflation of the renal parenchyma (pT3a) vs macroscopic inflation or peripelvic adipose tissue (pT3b).^20^, ^22^ However, that information could not be retrieved by the SEER database in our study. Additionally, as a prognostic factor, tumor size has the advantage of allowing pathologist agreement and accurate measurement through imaging techniques. A 3‐cm tumor size cutoff value is well validated as reflecting biological aggressiveness in bladder urothelial carcinomas, and the same cutoff value has been confirmed in UTUCs by Yan et al.^32^ Some retrospective studies suggested that tumors with PFI may be more likely to metastasize and contain more adverse features than tumors with RPI. As highlighted by Park et al,^33^ the renal parenchyma could be considered as a barrier to the systematic spread of UTUC. In addition, Park et al showed that tumors with RFI had more aggressive features than those with RPI, such as positive resection margins, higher tumor grades, and sessile tumor architecture.^34^ The current study demonstrated that the trend of OS benefits was consistent in patients with RPI or RFI. Our results showed that the effectiveness of chemotherapy is not related to the tumor size and pattern of invasion.

One limitation of this study is its retrospective design. Moreover, details of the treatment regimens, cycles, and sequences of chemotherapy were not reported in the SEER database. However, in some large population databases, the proportions of patients receiving neoadjuvant and adjuvant chemotherapy were 3%‐5.3% and 13%‐25%, respectively.^17^, ^19^ The survival benefit of neoadjuvant chemotherapy is mainly seen in patients achieving complete response^35^; complete response occurs in 38% of cases of bladder urothelial carcinoma^35^ and only 14% of UTUC cases.^36^ Moreover, as recommended by the EAU guidelines, the survival benefits of neoadjuvant chemotherapy for UUTC still need to be validated.^2^ According to the NCDB database, the proportion of patients with pT3‐4 or pN+ tumors receiving neoadjuvant chemotherapy is only 2.3%.^3^ We think that our outcomes will be relevant for patients with pT3N0M0 who are under preparation to receive adjuvant chemotherapy. Also not included in the SEER database were particular factors reflecting the aggressiveness of tumors (eg, tumor architecture and lymphovascular invasion). We therefore attempted to control only the observed risk factors with the IPTW‐adjusted propensity scoring technique, and acknowledge that prospectively designed studies are the best way to resolve this limitation. As previously mentioned, the SEER database did not grade tumors according to the World Health Organization/International Society of Urologic Pathologists (WHO/ISUP) coding system; however, the binary method of grading used (G1‐2 vs G3‐4) approximately correlates to the WHO/ISUP coding system (low vs high grade).^37^ It may be thought that data from 2005 to 2014 does not sufficiently reflect a contemporary study population. This time lag is unavoidable for a population‐based study, and is ameliorated by the fact that treatment of UTUCs staged as T3‐4 or N+ did not progress much during this time period, especially adjuvant treatment after radical nephroureterectomy.^38^

## 5 CONCLUSION

Chemotherapy may provide a significant OS benefits for patients with pT3N0M0 RPUC. The findings of our study could strengthen the evidence to use adjuvant chemotherapy for this rare group of patients.

## 6 Ethical approval and consent to participate

The data of our study were sought by the SEER database. So, the ethical approval is not necessary for our study.

We got approval from the National Cancer Institute to use data of patient from SEER database.

## CONFLICTS OF INTEREST

The authors declare that they have no competing interests.

## AUTHOR CONTRIBUTIONS

ZFL, JLH, and ZWL participated in study concepts and design; ZFL and HCW have contributed to data acquisition; ZWL and ZJX have contributed to quality control of data and algorithms; ZFL, JLH, and ZWL have contributed to data analysis and interpretation; JLH and JXZ have contributed to statistical analysis; ZFL, XDL, and YLY participated in manuscript preparation and editing; ZWL and JXZ have contributed to manuscript review.

## Supporting information

Table S1

## Data Availability

The datasets generated during the current study are available in the SEER database, https://seer.cancer.gov/data/.
