# Supplementary material for: The effectiveness of chemotherapy for patients with pT3N0M0 renal pelvic urothelial carcinomas: An inverse probability of treatment weighting comparison using Surveillance, Epidemiology, and End Results data
Source: Cancer Med. 2020 Jun 25;9(16):5756–66. doi: 10.1002/cam4.3238 (PMC7433845; doi:10.1002/cam4.3238)
Supplement: Supplementary file 1 — Table S1 [file CAM4-9-5756-s001.docx]

| **Confounding factors** | †**Covariates in each subgroup analysis** | **HR** | **95% CI** | ***P*** |
| --- | --- | --- | --- | --- |
| Age |  |  |  |  |
| <75 years | A | 0.681 | 0.496-0.933 | 0.017 |
| ≥75 years | A | 0.756 | 0.442-1.294 | 0.308 |
| Gender |  |  |  |  |
| Male | B | 0.687 | 0.491-0.962 | 0.029 |
| Female | B | 0.691 | 0.431-1.109 | 0.126 |
| Grade |  |  |  |  |
| G1-2 | C | 0.948 | 0.314-2.863 | 0.924 |
| G3-4 | C | 0.691 | 0.516-0.926 | 0.013 |
| Unknown | C | 0.316 | 0.101-0.989 | 0.048 |
| Patterns of invasion |  |  |  |  |
| RPI | D | 0.602 | 0.416-0.871 | 0.007 |
| RFI | D | 0.879 | 0.579-1.336 | 0.546 |
| Tumor size |  |  |  |  |
| ≤ 3cm | E | 0.615 | 0.371-1.022 | 0.060 |
| ＞3cm | E | 0.757 | 0.538-1.066 | 0.111 |
| Unknown | E | 0.523 | 0.142-1.919 | 0.328 |

**Supplementary Table 1. The effect of chemotherapy using multivariable COX regression in each subgroup**

Confounding factors: Age, Gender, Race, Lymph.node.removed, Grade, Insurance, Tumor size, Pattern of invasion; †Covariates in each subgroup analysis: A: adjusting the covariates in confounding factors except age;B: adjusting the covariates in confounding factors except gender; C: adjusting the covariates in confounding factors except grade; D: adjusting the covariates in confounding factors except pattern of invasion; E: adjusting the covariates in confounding factors except tumor size; ***RFI***: renal peripelvic/periureteral fat invasion; ***RPI***: renal parenchymal invasion
